# Variability in research ethics review of cluster randomized trials: a scenario-based survey in three countries

**DOI:** 10.1186/1745-6215-15-48

**Published:** 2014-02-05

**Authors:** Monica Taljaard, Jamie C Brehaut, Charles Weijer, Robert Boruch, Allan Donner, Martin P Eccles, Andrew D McRae, Raphael Saginur, Merrick Zwarenstein, Jeremy M Grimshaw

**Affiliations:** 1 Ottawa Hospital Research Institute, Clinical Epidemiology Program, 1053 Carling Avenue, Civic Campus, C409, Ottawa, ON K1Y 4E9, Canada; 2 Department of Epidemiology and Community Medicine, University of Ottawa, 451 Smyth Road, Ottawa, ON K1H 8M5, Canada; 3 Rotman Institute of Philosophy, Department of Philosophy, Stevenson Hall 2150, Western University, London, ON N6A 5B8, Canada; 4 Department of Medicine, University of Ottawa, Ottawa, ON Canada; 5 Department of Medicine, Western University, London, ON Canada; 6 Department of Epidemiology and Biostatistics, Western University, Kresge Building, Room K201, London, ON N6A 5C1, Canada; 7 Graduate School of Education and Statistics Department, Wharton School, University of Pennsylvania, 3700 Walnut Street, Philadelphia, PA 19104, USA; 8 Robarts Clinical Trials, Robartsca; 9 Institute of Health & Society, Newcastle University, 21 Claremont Place, Newcastle upon Tyne NE2 4AA, UK; 10 Department of Emergency Medicine, University of Calgary, Foothills Medical Centre, Calgary, AB Canada; 11 Division of Infectious Disease, Ottawa Hospital-Civic Campus, 1053 Carling Avenue, Ottawa, ON K1Y 4E9, Canada; 12 Centre for Health Services Sciences, Sunnybrook Health Sciences Centre, 2075 Bayview Avenue, Toronto M4N 3 M5, Canada; 13 Ottawa Hospital Research Institute, Clinical Epidemiology Program, The Ottawa Hospital - General Campus, 501 Smyth Road, Box 711, Ottawa, ON K1H 8 L6, Canada; 14 Ottawa Hospital Research Institute, Clinical Epidemiology Program, Ottawa Hospital, Civic Campus, 1053 Carling Avenue, Civic Box 693, Admin Services Building, ASB 2–004, Ottawa, ON K1Y 4E9, USA

**Keywords:** Cluster randomized trials, Informed consent, Research ethics guidelines, Research ethics review, Web-based survey

## Abstract

**Background:**

Cluster randomized trials (CRTs) present unique ethical challenges. In the absence of a uniform standard for their ethical design and conduct, problems such as variability in procedures and requirements by different research ethics committees will persist. We aimed to assess the need for ethics guidelines for CRTs among research ethics chairs internationally, investigate variability in procedures for research ethics review of CRTs within and among countries, and elicit research ethics chairs’ perspectives on specific ethical issues in CRTs, including the identification of research subjects. The proper identification of research subjects is a necessary requirement in the research ethics review process, to help ensure, on the one hand, that subjects are protected from harm and exploitation, and on the other, that reviews of CRTs are completed efficiently.

**Methods:**

A web-based survey with closed- and open-ended questions was administered to research ethics chairs in Canada, the United States, and the United Kingdom. The survey presented three scenarios of CRTs involving cluster-level, professional-level, and individual-level interventions. For each scenario, a series of questions was posed with respect to the type of review required (full, expedited, or no review) and the identification of research subjects at cluster and individual levels.

**Results:**

A total of 189 (35%) of 542 chairs responded. Overall, 144 (84%, 95% CI 79 to 90%) agreed or strongly agreed that there is a need for ethics guidelines for CRTs and 158 (92%, 95% CI 88 to 96%) agreed or strongly agreed that research ethics committees could be better informed about distinct ethical issues surrounding CRTs. There was considerable variability among research ethics chairs with respect to the type of review required, as well as the identification of research subjects. The cluster-cluster and professional-cluster scenarios produced the most disagreement.

**Conclusions:**

Research ethics committees identified a clear need for ethics guidelines for CRTs and education about distinct ethical issues in CRTs. There is disagreement among committees, even within the same countries, with respect to key questions in the ethics review of CRTs. This disagreement reflects variability of opinion and practices pointing toward possible gaps in knowledge, and supports the need for explicit guidelines for the ethical conduct and review of CRTs.

## Background

Cluster Randomized Trials (CRTs), that is, trials in which the units of randomization are intact social units or groups, rather than independent individuals, are an increasingly common design in health research [1,2]. It is convenient to distinguish between three kinds of CRTs depending on the level of intervention: cluster-cluster trials (experimental interventions administered to the entire cluster as a unit), professional-cluster trials (experimental interventions administered to health or other professionals), and individual-cluster trials (experimental interventions administered to patients or individuals) [3,4]. An example of a cluster-cluster trial is the COMMIT study, in which 22 communities in the United States and Canada were randomized to evaluate a community-wide advertising campaign to reduce smoking [5]. In the Nexus trial, an example of a professional-cluster trial, 247 primary care practices were randomized to evaluate the effect of audit and feedback and reminder messages on radiology referrals [6], while in the bed net trial, an example of an individual-cluster trial, 36 villages were randomized to evaluate the impact of distribution of insecticide-treated bed nets to individual citizens on malaria morbidity and mortality in remote areas of Cambodia [7].

There are many aspects of CRTs that complicate their *ethical* design, conduct and review (beyond the statistical complications) [3,8-10]. In the absence of guidance tailored to CRTs, researchers and research ethics committees have had to rely on variable interpretation of existing research ethics guidelines, which were developed primarily with individually randomized trials in mind. This has likely contributed to variation in how CRTs have been conducted in practice, as well as in the requirements and decisions by different research ethics committees [11,12]. Between 2008 and 2012, we conducted a mixed-methods research project to develop internationally accepted, principled guidelines for the ethical design, conduct, and review of CRTs [13]. As part of the effort to develop these guidelines, we conducted a web-based survey of chairs of research ethics committees (Research Ethics Boards (REBs) in Canada, Research Ethics Committees (RECs) in the United Kingdom, Institutional Review Boards (IRBs) in the United States). The main objectives of this survey were to a) evaluate the need among research ethics committees for guidelines specific to CRTs, b) gather information about the research ethics review process for CRTs at their institutions, and c) elicit their views on ethical issues in CRTs using a scenario-based approach.

## Methods

### Target population

The target population was chairs of research ethics committees (one per institution) in Canada, the United States, and the United Kingdom that a) are currently active, b) review health-related human subjects (that is, biomedical) research, and c) review randomized controlled trials.

### Sample size

The target sample size for the survey was 300, based on estimating the proportion of chairs who agree or strongly agree that there is a need to develop research ethics guidelines for CRTs. A total of 300 respondents would be sufficient to allow a 95% two-sided confidence interval to extend ± 5.7% around an expected proportion of 50%, which is the proportion that yields the maximum width. Assuming a response rate of 50%, we anticipated that we would need to survey all REBs in Canada, all RECs in the United Kingdom, and a random sample of IRBs from the United States. Our expected sample sizes were therefore 90 to 100 Canadian chairs, 50 to 60 United Kingdom chairs, and 150 to 160 United States chairs, for an overall total of 300 respondents.

### Sampling strategy

**Figure 1:**
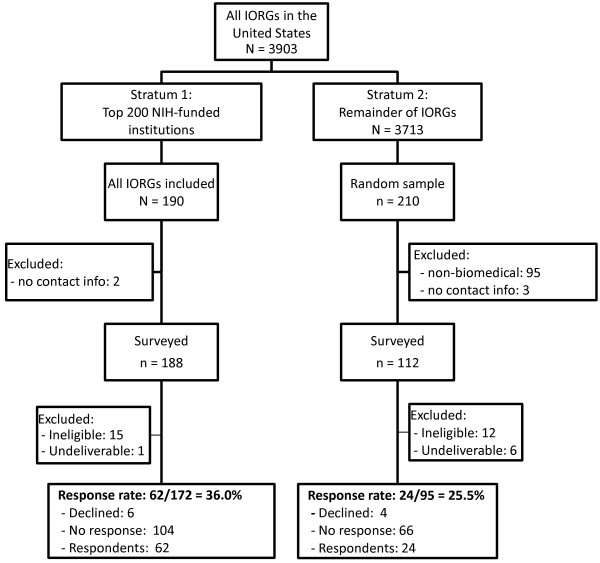
**Flow diagram summarizing the identification and inclusion of Institutional Review Boards (IRBs) in the United States.** IORGs, Institutional Review Board Organization; NIH, National Institutes of Health.

**Figure 2:**
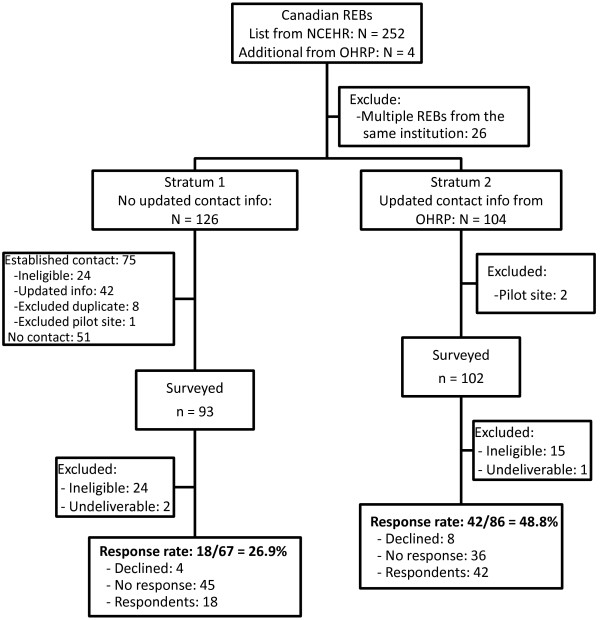
**Flow diagram summarizing the identification and inclusion of Research Ethics Boards (REBs) in Canada.** NCEHR, National Council on Ethics in Human Research; OHRP, Office of Human Research Protections.

**Figure 3:**
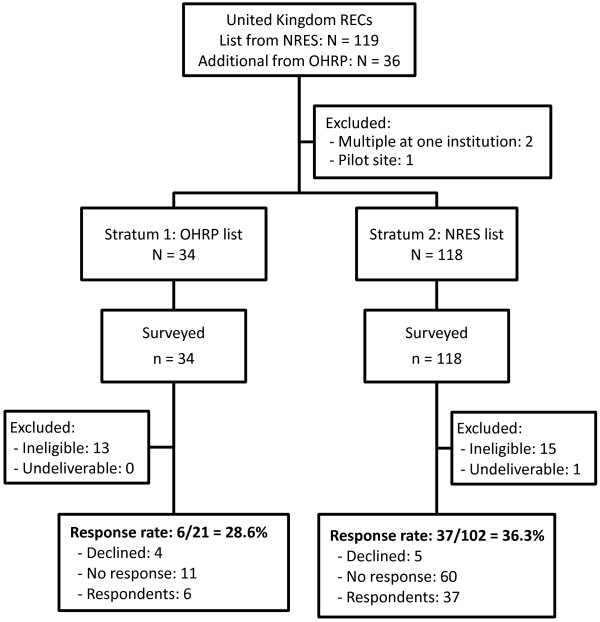
**Flow diagram summarizing the identification and inclusion of Research Ethics Committees (RECs) in the United Kingdom.** NRES, National Research Ethics Service; OHRP, Office of Human Research Protections.

Obtaining an adequate sampling frame for research ethics committees in the three countries was challenging. Figures 1, 2 and 3 present flow diagrams summarizing the identification, sampling, and participation of research ethics chairs in the United States, Canada, and the United Kingdom. In the United States, we used a list of 3,903 so-called IORGs ('Institutional Review Board Organizations’) provided by the Office of Human Research Protections (OHRP). This list did not differentiate between biomedical or nonbiomedical IRBs. To increase the efficiency of sampling, we separated the IORGs into two strata: the first stratum consisted of 190 IORGs successfully matched to the top 200 National Institutes of Health funded institutions [14] (and thus more likely to be biomedical); the second consisted of the remainder of the IORGs, from which we randomly sampled 210. Among these, we used online searches to exclude those that were clearly nonbiomedical. The final sample size in the United States was 300.

In Canada we used a list of 252 'biomedical’ REBs supplied by the National Council on Ethics in Human Research (NCEHR). As this list was approximately 10 years out of date, it was cross-checked against the list of Canadian-based IORGs provided by the OHRP to obtain updated contact information and to identify any additional REBs. For those REBs not on the OHRP list, we attempted to obtain their updated contact information by conducting online searches, sending an email to the contact on file, and making telephone calls. The final sample size in Canada was 195 REBs.

In the United Kingdom, we used a list obtained from the National Research Ethics Service (NRES) to identify 119 active RECs. We cross-checked this list against the United Kingdom-based IORGs on the list provided by the OHRP and identified an additional 36 (mostly university-based) RECs. The final sample size in the United Kingdom was 152 RECs.

### Survey questionnaire

After pre-testing [15], the final questionnaire consisted of 37 items and required 30 to 45 minutes to complete. The first section of the questionnaire presented three scenarios of CRT protocols that (hypothetically) had been submitted at the chair’s institution. The scenarios are presented in Table 1. Briefly, the first scenario was a cluster-cluster trial to evaluate a mass media advertising campaign to increase colorectal cancer screening; the second was a professional-cluster trial of an educational intervention targeted at general practitioners (GPs) to reduce imaging for nontraumatic back and knee pain; the third was an individual-cluster trial randomizing villages in Cambodia to evaluate effectiveness of insecticide-treated bed nets against malaria. The questions following each scenario addressed the type of review the study would undergo at their institution (full review, expedited review, no review, or don’t know) and which cluster members they would consider to be research subjects. Options for research subjects varied in each scenario from more inclusive (for example, all cluster members), to less inclusive (for example, only those who received an intervention or who participated in data collection). Open text boxes were provided to allow respondents to explain their answers. Additional questions addressed informed consent procedures, and benefits and harms, and will be reported elsewhere. The second section of the questionnaire addressed the perceived need for ethics guidelines for CRTs and included space to make any other comments about ethical issues in CRTs. A final section requested demographic and descriptive information.

**Table 1:** Description of three scenarios of cluster randomized trials presented in survey

**Scenario**	**Description**
**Cluster-cluster trial (mass media advertising for colorectal cancer screening)**	A researcher at your institution is proposing a cluster randomized trial to evaluate a radio, television, and billboard advertising campaign aimed at increasing the proportion of community residents who receive colorectal cancer screening according to well-accepted national guidelines. Cluster randomization is used because the intervention (the advertising campaign) is delivered to the community as a whole. Twenty cities will be randomly assigned to either the intervention group or a control group receiving no intervention. A random sample of 500 residents in each city will be surveyed before the intervention, and a separate random sample of 500 residents in each city will be surveyed after the intervention, to determine the proportions that have been screened. The surveys will be conducted by telephone using random digit dialing. No personally identifying information will be collected in the surveys.
**Professional-cluster trial (professional education to reduce imaging)**	A researcher at your institution is proposing a cluster randomized trial to evaluate an educational intervention designed to reduce unnecessary requests for X-rays in accordance with well-accepted national guidelines (which have been widely available for two years) for patients with nontraumatic back and knee pain. All 250 general practices in the study area will be randomly assigned to either the educational intervention or a control group. The guidelines will be mailed to all general practitioners (GPs) in intervention and control practices, but intervention GPs will additionally receive regular reminder messages about the guidelines by mail, as well as feedback about the number of X-rays ordered by their whole practice compared with requests made by all GPs in the study. Study outcome 1 is the number of X-rays ordered per thousand patients using data routinely collected by radiology departments. These data will be sent to the researchers with GP identifiers included, to allow the researchers to prepare feedback to the practices about their number of X-ray requests. Study outcome 2 is the percentage of X-ray requests that are concordant with the guidelines, determined by researchers reviewing and collecting anonymized data from a randomly chosen subset of 100 patient records per practice.
**Individual-cluster trial (distribution of bed nets against malaria)**	A researcher at your institution is proposing a cluster randomized trial to evaluate a malaria prevention intervention. Thirty villages in Cambodia with a total population of 10,000 will be randomly assigned to either an intervention group in which insecticide-treated bed nets will be distributed to all residents (by delivering them to each household), or a control group in which no bed nets will be distributed to any residents. Cluster randomization is used because the bed nets can only be effective in preventing the spread of malaria if they are used by the majority of residents in a village and because the investigators feel that it would not be acceptable to distribute bed nets to only a random half of the residents in a village. Although insecticide-treated bed nets have previously been shown to be effective against malaria in most tropical and subtropical regions, there are differences in vector biting cycles and malaria epidemiology in South East Asia that raise questions about the effectiveness of the insecticide-treated bed nets in Cambodia. Village volunteers in both intervention and control villages will be trained to recognize malaria symptoms and administer standard anti-malarial treatment. Villagers will be told that they can consult the village malaria worker when unwell. Malaria prevalence will be determined before and after the intervention using blood tests from cross-sectional random samples of 250 people per village.

### Survey implementation

The survey was conducted between April and July 2011. A series of five contacts, based on Dillman’s recommendations for the implementation of mail and internet surveys, was used [16]. An invitation was sent to the chair or committee contact on file including the survey URL, a unique password, and details of confidentiality. A thank you and reminder email was sent to all contacts 1.5 weeks after the invitation, with reminder emails sent 3 and 5 weeks after the invitation. As a 'special’ contact has been shown to improve overall response to mail surveys, a final reminder was sent by postal mail approximately 8 weeks after the initial invitation. A $30 Amazon gift certificate was offered to each respondent as an incentive.

### Data analysis

Categorical variables were described using frequencies and percentages. Exact or asymptotic methods were used to calculate 95% confidence intervals (CIs) of percentages for the main outcomes. We conducted tests of association using chi-squared or Fisher’s exact tests as appropriate. Verbatim comments from open text boxes were selected to illustrate the range of perspectives.

### Ethics approval

Participants were informed that participation is voluntary and assured of the confidentiality of their responses. Submission of the questionnaire was considered as consent. The study procedures were approved by the Ottawa Hospital Research Ethics Board.

## Results

### Response rates

Of 647 contacts surveyed, 105 were discovered to be ineligible; of the remaining 542 chairs, 189 responded, giving an overall response rate of 35%. Response rates were slightly higher in Canada (39%) than in the United Kingdom (35%) and the United States (32%) (*P* = 0.36). To help understand why response rates were lower than anticipated, we compared response rates by various aspects of the sampling design. First, we explored whether outdated contact information was a factor within Canada. We found that response rates were significantly higher among those with updated contact information from the OHRP list (49% versus 27%, *P* = 0.006). Next, we explored whether the inclusion of nonbiomedical committees was a factor within the United States. We found that response rates were higher among the top funded NIH institutions (more likely to be biomedical) (36% versus 26%, *P* = 0.08). In the United Kingdom, we compared response rates among university (29%) and National Research Ethics Service committees (36%) but found no significant difference (*P* = 0.50).

### Characteristics of respondents

Table 2 presents characteristics of participating ethics committees and chairs in the three countries. There were some notable differences among countries: participating committees in the United States tended to be in existence longer than those in the United Kingdom (85% established before 1990 in the United States versus only 27% in the United Kingdom). Respondents in the United States reviewed a median of 30 protocols per month, compared to 5 and 8 in Canada and the United Kingdom respectively. Approximately two-thirds of respondents in Canada and the United Kingdom reported that they reviewed at least one CRT per year, compared to 92% of respondents in the United States. Only three committees (all in the US) indicated that they had guidelines in place for CRTs, and only eight in total indicated that they were aware of any guidelines for CRTs.

**Table 2:** Characteristics of participating ethics committees and chairs

	**Canada**	**United States**	**United Kingdom**	**Total**
	**(N = 60)**	**(N = 86)**	**(N = 43)**	**(N = 189)**
Response rate	39.2% (60/153)	32.3% (86/266)	35.0% (43/123)	34.9% (189/542)
**Ethics committee characteristics**				
**Year established**				
<1990	23 (42.6)^a^	66 (84.6)	10 (27.0)	99 (58.6)
1990 to 1999	14 (25.9)	9 (11.5)	13 (35.1)	36 (21.3)
> = 2000	17 (31.5)	3 (3.9)	14 (37.8)	34 (20.1)
# Protocols/month (Median, Q1-Q3)	5 (3 to 20)	30 (10 to 60)	8 (7 to 8)	10 (5 to 37.5)
**Type of committee**^b^				
University	18 (30.0)	49 (57.0)	8 (18.6)	75 (39.7)
Hospital/healthcare	41 (68.3)	41 (47.7)	-	82 (56.2)
National Research Ethics Service	-	-	31 (72.1)	31 (16.0)
Other nonprofit	2 (3.33)	9 (10.5)	0	11 (5.8)
Other for-profit	5 (8.3)	2 (2.3)	0	7 (3.7)
**Number of CRTs /year**				
None	18 (32.7)	3 (8.1)	27 (34.6)	48 (28.2)
1 to 5	35 (63.6)	28 (75.7)	38 (48.7)	101 (59.4)
>5	2 (3.6)	6 (16.2)	13 (16.7)	21 (12.3)
Have guidelines in place for CRTs	0	3 (3.8)	0	3 (1.7)
Aware of guidelines for CRTs	1 (1.8)	3 (3.8)	4 (10.8)	8 (4.7)
**Ethics Chair characteristics**				
**Years experience as chair**				
<2	9 (16.4)	12 (15.2)	9 (24.3)	30 (17.5)
2 to 5	27 (49.1)	28 (35.4)	12 (32.4)	67 (39.2)
>5	19 (34.5)	39 (49.3)	16 (43.2)	74 (43.3)
**Years experience as member**				
<6	26 (47.3)	24 (30.4)	13 (35.1)	63 (36.8)
6 to 10	18 (32.7)	21 (26.6)	16 (43.2)	55 (32.2)
>10	11 (20.0)	34 (43.0)	8 (21.6)	53 (31.0)
**Highest degrees**^c^				
Doctorate	24 (43.6)	36 (45.0)	17 (46.0)	77 (44.8)
Medical	23 (41.8)	43 (53.8)	13 (35.1)	79 (45.9)
Masters	16 (29.1)	10 (12.5)	11 (29.7)	37 (21.5)
Undergraduate	4 (7.3)	4 (10.8)	2 (2.5)	10 (5.8)

^a^ Table entries represent number (%) unless otherwise indicated.

^b^A committee can be classified as more than one type.

^c^A chair can indicate more than one degree.

CRT, cluster randomized trial.

### Perceived need for guidelines

Table 3 presents respondents’ perceived need for ethics guidelines. Overall, 144 chairs (84%, 95% CI 79 to 90%) agreed or strongly agreed that there is a need for ethics guidelines for CRTs, 158 (92%, 95% CI 88 to 96%) that research ethics committees could be better informed about distinct ethical issues surrounding CRTs, and 52 (31%, 95% CI 24 to 38%) that ethics application forms ought to be designed separately for the CRT design. These responses were not significantly different among the three countries (results not shown). They were also not significantly associated with experience reviewing CRTs: agreement with the need for guidelines was 84% versus 85% (*P* = 0.82), and agreement that committees could be better informed 98% versus 91% (*P* = 0.10) among committees with and without prior experience reviewing CRTs, respectively.

**Table 3:** Need for ethics guidelines for cluster randomized trials (CRTs): number (%) agreeing or strongly agreeing with each statement

	**Canada**	**United States**	**United Kingdom**	**Total**
	**(N = 55)**	**(N = 79)**	**(N = 37)**	**(N = 175)**
There is a need to develop ethics guidelines for CRTs	45 (81.8)	67 (84.8)	32 (86.5)	144 (84.2)
Ethics committees need more information about ethical issues in CRTs	52 (94.6)	73 (92.4)	33 (89.2)	158 (92.4)
Application forms ought to be designed separately for CRTs	19 (35.2)	24 (30.8)	9 (24.3)	52 (30.8)

### Types of review required for each scenario

**Figure 4:**
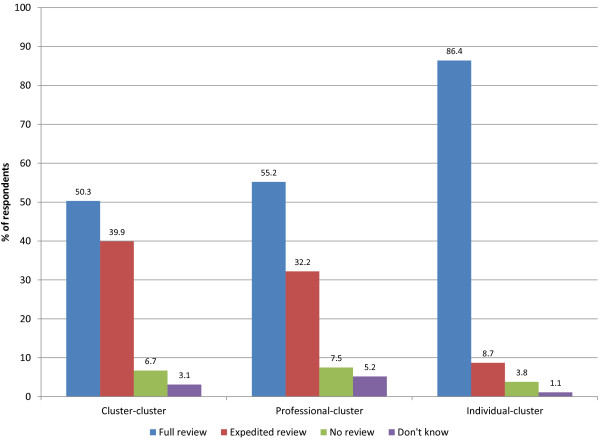
Type of review indicated by respondents for the cluster-cluster, professional-cluster, and individual-cluster scenarios.

Figure 4 presents the type of review that would be required for each scenario. There was large variability among respondents in two of the three scenarios. In particular, for the cluster-cluster (mass media campaign to increase colorectal cancer screening) and professional-cluster (education of GPs to reduce imaging) scenarios, approximately half of respondents indicated that these studies would need to undergo full review. This variability persisted even within countries: for the cluster-cluster scenario, the percentages indicating full review in Canada, the United Kingdom and the United States were 55%, 57%, and 42%, respectively, and for the professional-cluster scenario, 65%, 51%, and 48%. On the other hand, respondents were largely in agreement in the individual-cluster scenario (bed nets to prevent malaria), with 86% of respondents indicating that a full review would be required (88%, 87% and 85% in Canada, the United Kingdom, and the United States, respectively). Respondents were asked to explain their answers and selected explanations in support of each type of review are presented in Tables 4 and 5, by country, for the cluster-cluster and professional-cluster scenarios. These responses illustrate the diversity of views even within the same countries.

**Table 4:** Illustrative verbatim explanations for the type of review required, by country: cluster-cluster scenario

**Country**	**Type of review**	**Explanation**
Canada	Full review	Full Board review would be recommended for the following reasons: a) community consent versus individual consent, b) there is an element of deception, c) the treatment/intervention received is determined by randomization, d) the telephone surveys deal with potentially sensitive topic (colorectal screening) and could result in situations where there is a need for follow-up medical and/or psychological care.
	Expedited	Review is necessary due to 'research’ component and to ensure privacy and patient rights are protected. Expedited as there is no experimental manoeuvre, screening is according to national guidelines.
	No review	The researcher is outside our jurisdiction - They are using subjects who are unlikely to be our patients. At the beginning, consent is not required except at the municipal level. There is no financial liability to the hospital. There is no treatment intervention. I see this as no different from the telephone surveys I receive now at home. It would be polite and professionally correct to bring this to the ethics committee as an FYI. If the researcher wanted to add gravitas to his/her survey they might ask for the 'blessing’ of the ethics committee. However , as before, it not being done on hospital property, involves no patients and no therapeutic intervention.
United Kingdom	Full review	The exceptions are minimal risk studies with small numbers of participants, which have no contentious issues. In this case, 1,000 participants, approached by random 'cold-call’ telephone with verbal consent required and no prior information, would be rated by us as large and potentially contentious.
	Expedited review	The proposed trial deals with a situation where the normal practice is 'no intervention’; there’s no specific bowel screening promotion as described in the UK. Expedited review would be to address whether the materials provided to the Mayors/Officials of the cities involved enough information to understand the proposed research. There is low/no risk to participating citizens and the interventions would be considered 'light touch’ and of low risk; unless of course review of the proposed telephone interview guidelines revealed significant intrusive or alarming questions.
	No review	Because it is the effectiveness of the advertising campaign that is to be evaluated and this in itself is not a medical intervention and I would regard the study as in support of delivering an established standard of normal care.
United States	Full review	I think the questions raised by the methodology, in particular the randomization to a community intervention without individual consent, would merit consideration by the full board.
	Expedited review	The study as described presents no more than minimal risk to the sample involved. The dependent measure is related to behavior. The study is not inviting participants to get a colorectal exam offered by the investigators. The study measures are related to the effectiveness of an information campaign.
	No review	This is a survey, should be exempt 45 CFR 46.101 #2.

**Table 5:** Illustrative verbatim explanations for the type of review required, by country: professional-cluster scenario

**Country**	**Type of review**	**Explanation**
Canada	Full review	This study would require REB approval because there is an intervention, patient data is collected and consent is not planned. There is the onus to review fully given the lack of a consent process, regardless of the reason.
	Expedited	Meets all Tri-Council Policy Statement (TCPS) criteria for minimal risk.
	No review	This seems to be more akin to a Quality Improvement study of whether an educational intervention and reminders about an accepted Clinical Guideline leads to greater acceptance and adoption of these practices. Our REB does not review these, but there is a process of review and support for these studies through our Quality and Risk Management Dept. All interventions and practices are clinically indicated and data is only aggregate.
United Kingdom	Full review	Because of the randomisation of practices. Otherwise could have been considered as a service evaluation and therefore not require review (potentially!)
	Expedited review	Minimal risk, using routinely collected data, assuming that patient data for outcome 2 are properly anonymised and secure, and do not require information that is outside normal clinical care.
	No review	This is classic improvement methodology and would not need review.
United States	Full review	There is an issue of physicians being known to the researchers and the potential for data about their behavior that could be used to potentially harm the physician’s reputation due to information about his/her medical practices (x-rays).
	Expedited review	We would not consider an educational intervention as treatment. The educational intervention mailing established imaging guidelines is clearly minimal risk. Measuring radiology imaging by GP is not private information and routinely measured by many external entities. The outcome measures of number X-rays/thousand and anonymized medical records review would meet Expedited review categories.
	No review	This seem to be primarily a trial to determine quality of care and use or misuse of diagnostic studies. No patient is harmed or helped by the study. It may help cost containment. It doesn’t need to come to the IRB.

### Identification of research subjects

The identification of research subjects is presented in Table 6, sorted from most to least inclusive. In the cluster-cluster scenario (mass media campaign to increase colorectal cancer screening), there was considerable uncertainty as to who would be considered research subjects. Although the vast majority classified participants in the telephone surveys as research subjects, there was uncertainty whether all residents, only those exposed to the advertisements, or only those screened were research subjects. Fewer than one-third of respondents said that they would consider all city residents (intervention and control arms) to be research subjects. A selection of verbatim quotes supporting more inclusive views is:

1. Canada: They are all involved. The degree of protections required depends on the protocol itself, potential harms or burdens, potential benefits, what would happen anyway and what happens specifically because of the research. All this will vary.

2. United Kingdom: The intervention is targeted at the entire community, not only those who undergo screening.

3. United States: All residents in the intervention cities are potentially exposed to effects in the information campaign, and thus should be protected from effects of inappropriate content, no matter whether they actually were exposed to the advertisements. (For example, a spouse might hear the advertisement and prompt the other spouse to schedule screening.)

**Table 6:** Identification of research subjects in the cluster-cluster, professional-cluster, and individual-cluster trial scenarios

	**Frequency (%)**	
**Cluster-cluster trial**	**Yes**	**No**
All city residents in the intervention arm	50 (29.8)^a^	118 (70.2)
All city residents in the control arm	46 (27.9)^a^	119 (72.1)
Residents exposed to the advertisements	68 (39.5)^a^	104 (60.5)
Residents in intervention arm receiving screening	72 (42.1)^a^	99 (57.9)
Residents participating in the telephone survey	171 (90.0)^a^	19 (10.0)
**Professional-cluster trial**	**Yes**	**No**
*Patient-level:*		
All patients in the practices	37 (22.6)	127 (77.4)^a^
Patients presenting with back or knee pain	63 (38.4)	101 (61.6)^a^
Patients providing data for study outcome 1	64 (38.6)	102 (61.5)^a^
Patients providing data for study outcome 2	104 (62.3)^a^	63 (37.7)
*Professional-level:*		
All GPs in intervention practices	109 (66.9)^a^	54 (33.1)
All GPs in control practices	103 (63.6)^a^	59 (36.4)
GPs whose patients present with back or knee pain	127 (77.4)^a^	37 (22.6)
**Individual-cluster trial**	**Yes**	**No**
Residents receiving bed nets	158 (88.3)^a^	21 (11.7)
Residents in control arm receiving no bed nets	139 (79.9)^a^	35 (20.1)
Residents providing blood samples	177 (98.3)^a^	3 (1.7)
Village malaria workers	58 (36.7)	100 (63.3)^a^

^a^Indicates that an answer is in agreement with the Ottawa Statement. GPs, general practitioners.

Examples of less inclusive views are:

1. Canada: No data is collected from all residents, only those sampled. Advertising for clinical procedures with or without generic advice is a normal experience of everyday life.

2. United Kingdom: The intervention is the advertising so those not exposed and not questioned are no different to those citizens in any uninvolved city. The screening itself is not research;- it is a standard health process which a proportion of citizens would choose to undergo regardless.

3. United States: The entire population cannot be considered as research subjects, both from a practical and study design perspective. Some may be affected by the advertising, but there are myriad ways for individuals to encounter similar messages and it would be virtually impossible to know what influenced their ultimate behavior. The residents who are directly contacted can more legitimately be considered as 'research’ subjects, given their direct participation in the survey instruments.

In the professional-cluster scenario (GP education to reduce imaging), there was a similar degree of uncertainty. Approximately two-thirds of respondents indicated that they would consider GPs to be research subjects (intervention and control arms). Nearly two-thirds indicated that patients whose medical records are reviewed and nearly 40% that patients whose aggregated data are used to calculate imaging rates are research subjects; fewer than one-quarter indicated that they would consider all patients as research subjects. A selection of illustrative quotes that support more inclusive views are:

1. Canada: All GPs in the intervention and control practices even if their own habits aren’t being collected, because results will reflect on the quality of the practice in general and thus may impact on all of the GPs practicing in that practice.

2. United States: Would be concerned about whether study would have an impact on ordering of x-rays overall, so even patients who present with other than back or knee pain could be affected.

3. United Kingdom: Even if anonymised aggregated data is collected technically the patients are research subjects and the ethics committees are acting on their behalf to protect the appropriate use of their data.

Examples of less inclusive views are:

1. United Kingdom: This trial is concerned with GPs - the aim is simple - if given feedback will GPs reduce the number of X ray requests - patients are not subjects - they are just a source of data.

2. United States: The patients are not having an intervention performed on them directly and are therefore not research subjects. The physicians are being active participants and are therefore research subjects.

In the individual-cluster scenario (bed nets to prevent malaria) there was much less variability. Almost all chairs indicated that residents who provided blood samples (98%), and the majority of those who received bed nets (88%), would be considered research subjects. A slightly lower percentage (80%) would also consider residents in control communities (not receiving bed nets) to be research subjects. Approximately 40% included village malaria workers as research subjects.

### Any other comments

Several respondents identified a concern with respect to cluster-level interventions that may be considered as experimental interventions at the individual level; they were concerned that such studies may lack an appropriate rationale for the adoption of the CRT design, and that it may be an attempt by researchers to sidestep conventional informed consent requirements. These concerns are included below as verbatim quotes:

1. Canada: Occasionally I have been aware of cluster trials that deal with the evaluation of different forms of practice that are currently being done and for which there are no current standards of care proscribed. For example, use of different sets of antibiotics after cardiac interventions or changes in feeding in ICUs. What has been questioned are the following: What really constitutes an intervention (using (Tri-Council Policy Statement) as a guide) and therefore when should consent be obtained; where would one draw the line between quality assurance and research? Concern has also been raised about whether the trend for cluster trials is a mechanism to avoid the rigors of the consent process. Are letters of information sufficient to address this concern. These issues relate not so much to the large population studies, but rather to cluster trials being conducted in the current health care settings.

2. Canada: Most of the discussions we have had when discussing cluster RCT centered around the informed consent issues, when to waive consent, how much information to reveal, how much choice is there when the whole institute is already randomized to a certain intervention or not, and how to address situations where it is not beneficial for certain patients to be enrolled in the wrong intervention for their situation. For example, in a hospital cluster RCT where one hospital is randomized to a certain surgical process while the other is not, patients who are contraindicated to that surgery who end up in the intervention arm should be given the opportunity to refuse participation. In which case, they may have to be referred to another hospital not in the study.

Another identified concern was determining whether a study constitutes research as opposed to program evaluation or quality improvement:

1. United States: We have struggled with many issues related to this- when the Ministry of a country plans a roll out of a public health intervention and the research is to perform a cluster analysis of the intervention, what is research, what is a public health program evaluation and similar issues. This is still a topic of discussion and debate, especially between the IRB and international researchers.

## Discussion

This survey was conducted as part of a five-year project studying ethical issues in CRTs [13]. Following a series of empirical studies (including this survey), an in-depth ethical analysis, and an extensive consensus process, the Ottawa Statement ─ the first comprehensive ethics guidelines specific to CRTs ─ was published in November 2012 [17]. Although the survey was fielded before development of the Ottawa Statement, it is informative to interpret the results of the survey in light of the recommendations in the Ottawa Statement.

The first aim of the survey was to determine the need for research ethics guidelines for CRTs among research ethics chairs internationally. We found that 84% of research ethics chairs overall recognized the need for ethics guidelines for CRTs and 92% the need for education of ethics committees about ethical issues in CRTs. The perceived need for guidelines was not significantly associated with experience reviewing CRTs, which suggests that the need remains widespread and is not declining with experience. In our survey of 182 trialists (authors of CRTs) conducted as part of the five-year project, 74% (95% CI 67 to 80%) agreed or strongly agreed that there is a need to develop ethics guidelines for CRTs and 70% (95% CI 63 to 77%) that ethics committees could be better informed about distinct ethical issues surrounding CRTs [12]. Surprisingly, the proportions of research ethics chairs indicating the need for ethics guidelines were higher than the corresponding proportions of trialists.

Our second aim was to investigate research ethics review procedures for three different kinds of CRTs. We found that there was little agreement among committees in the type of ethics reviews required in the cluster-cluster and professional-cluster scenarios, both among and within countries. Whereas regulatory differences may explain differences among countries, it does not explain within-country variability. Whereas there was more agreement in the individual-cluster scenario, cluster-cluster trials and professional-cluster trials often evaluate public health programs or quality improvement initiatives, and it is not always clear if such trials ought to be considered research and undergo research ethics review. According to Recommendation 1 in the Ottawa Statement, all CRTs involving human research participants must be submitted to a research ethics committee. This includes CRTs evaluating quality improvement and knowledge translation interventions, and those in education or public health research. The type of ethics review is subject to the discretion of the committee; research ethics committees ought to undertake a proportional approach to the review of study protocols such that CRTs that pose substantial risk or involve vulnerable participants ought to receive intensive scrutiny, whereas CRTs that pose low risk and do not involve vulnerable participants may be eligible for an expedited or delegated review. Although the majority of respondents in all three scenarios agreed that some type of review is required (as opposed to no review), variability in the type of review required is an important finding as it reflects fundamental differences among committees with respect to the perceived level of risk associated with each scenario and the perceived vulnerability of those who might be considered participants. The type of review may have implications with respect to the level of scrutiny a protocol will receive during the review process, the number of reviewers assessing a protocol, and the time required to complete the review process.

Our third aim was to characterize the views of research ethics chairs on the identification of research subjects. The proper identification of research subjects is of considerable importance, as only those cluster members who are research subjects properly fall under the protection of research ethics committees and the regulations under which they operate. We observed substantial variability among chairs in all three countries with respect to the identification of research subjects in the cluster-cluster and professional-cluster scenarios. The individual-cluster scenario, which most resembles a standard randomized controlled trial, presented less disagreement. According to the Ottawa Statement [17,18] a research participant is defined as any individual whose interests may be affected as a result of study interventions or data collection procedures; that is, any individual who is the recipient or the direct target of a study intervention (whether an individual level or cluster level intervention), with whom researchers interact for study purposes, or about whom identifiable private information is collected. In the cluster-cluster scenario, fewer than one-third of chairs would consider residents in intervention and control communities to be research subjects, even though study interventions are targeted at them. It is a unique characteristic of CRTs that there can be participants at both individual and cluster levels. Surprisingly, in agreement with the Ottawa Statement, two-thirds of chairs indicated that they would consider health professionals to be research subjects. On the other hand, nearly 40% would consider patients whose health professionals participate in a knowledge translation intervention and who contribute only aggregate data (that is, practice-level imaging rates) to be research subjects. It is possible that those respondents who were broadly inclusive in identifying individuals who may be subjects in a CRT were doing so because they believed that the study required full ethics review and this would be one way to that end. Furthermore, although the need to seek informed consent is a separable issue [19], it is possible that chairs conflated the identification of research participants with the need to seek informed consent. The Ottawa Statement may help to avoid conceptual confusion by providing clarity on these important questions.

To the best of our knowledge, this is the first study to compare deliberations and decisions among research ethics committees in the review of CRTs. Although the scenarios were hypothetical, they represent three common settings where CRTs are used and are based on published CRTs. Participants in our survey provided perspectives from chairs in three countries: Canada, the United States, and the United Kingdom, and represented a variety of institutions: universities, hospitals, non-profit and for-profit, and varying degrees of experience. Our study provides empirical data of variability among committees both within and among countries in key decisions about the ethics review and conduct of CRTs. Although variability among ethics committees is not necessary morally problematic [20], these decisions have clear repercussions for the ability of research ethics committees to fulfill their purpose which includes protecting the rights and wellbeing of all research participants and to provide independent, competent, and timely review of the ethics of proposed studies [21].

Our study has two main limitations. First, our response rate was low and results may therefore not be representative. There can be many reasons for the low response. One is that our sampling frame was imperfect. Despite our best efforts at obtaining updated contact information and screening out ineligible committees prior to sampling, 105 of the 647 sampled committees (16%) either indicated that they were ineligible (that is, were nonbiomedical or did not review randomized controlled trials) or had invalid email addresses. Our stated response rate is conservatively calculated, meaning that all non-respondents are assumed to be eligible and included in the denominator of the response rate. The type of contact information varied: in some cases we had an email address for a named chair, while for others, only a central contact was available. Our analyses of response rates showed that response rates were higher among committees with more recent contact information; it was also higher among committees at NIH-funded institutions (more likely to be biomedical). Finally, our survey was relatively long and presented three complex scenarios; research ethics chairs are busy professionals and may simply not have had the time to complete the survey. Although we do not have any information that would allow us to investigate differences between respondents and non-respondents, we have no reason to expect answers of non-respondents to differ substantially from those observed in this survey.

A second limitation is that the descriptions of the scenarios were necessarily brief. In practice, ethics committees would have access to the full study protocol. It is possible that at least some of the variability observed is due to the lack of clarity or incompleteness of the information provided. However, we believe that the most relevant information was presented in each scenario, and the results are in line with findings from our survey of CRT investigators, which suggested that there are substantial challenges in practice. We were pleasantly surprised by the level of detail and thoughtfulness put into the open text responses. It was apparent that the majority of respondents paid careful consideration to the details provided in the scenarios and presented well-articulated and clear responses. This suggests that respondents understood that the scenarios were intended to trigger reflection on a certain class of study, rather than limit their consideration to those details provided in the survey alone.

## Conclusions

CRTs are an important research design, but research ethics committees and investigators need guidance as to ethically appropriate practices. Our scenario-based survey provides empirical evidence of the likely variability of opinion and practices within and among countries, pointing towards possible gaps in knowledge, and further supporting the need for guidelines.

## Abbreviations

CRT: cluster randomized trial; GP: general practitioner; IORG: Institutional Review Board Organization; IRB: Institutional Review Board; NCEHR: National Council on Ethics in Human Research; NIH: National Institutes of Health; NRES: National Research Ethics Service; OHRP: Office of Human Research Protections; REB: Research Ethics Board; REC: Research Ethics Committee; TCPS: Tri-Council Policy Statement.

## Competing interests

RS is the chair of the Ottawa Hospital Research Ethics Board. While conducting the survey, the authors were involved in the development of ethics guidelines for cluster randomized trials.

## Authors’ contributions

MT, JCB, CW, RB, AD, MPE, ADM, RS, MZ, and JGM participated in the conception and design and acquisition of data. MT conducted the analysis and drafted the manuscript. MT, JCB, CW, RB, AD, MPE, ADM, RS, MZ, and JGM contributed to interpretation of data, revised the manuscript critically for important intellectual content, and all authors read and approved the final manuscript.
